# Mental Health in COVID-19 Pandemic: A Meta-Review of Prevalence Meta-Analyses

**DOI:** 10.3389/fpsyg.2021.703838

**Published:** 2021-09-21

**Authors:** Geovan Menezes de Sousa, Vagner Deuel de Oliveira Tavares, Maria Lara Porpino de Meiroz Grilo, Monique Leite Galvão Coelho, Geissy Lainny de Lima-Araújo, Felipe Barreto Schuch, Nicole Leite Galvão-Coelho

**Affiliations:** ^1^Laboratory of Hormone Measurement, Department of Physiology and Behaviour, Federal University of Rio Grande Do Norte, Natal, Brazil; ^2^Graduate Program in Psychobiology and Department of Physiology and Behaviour, Federal University of Rio Grande Do Norte, Natal, Brazil; ^3^Brain Institute, Federal University of Rio Grande Do Norte, Natal, Brazil; ^4^Department of Sports Methods and Techniques, Federal University of Santa Maria, Santa Maria, Brazil; ^5^National Science and Technology Institute for Translational Medicine (INCT-TM), São Paulo, Brazil; ^6^NICM Health Research Institute, Western Sydney University, Westmead, NSW, Australia

**Keywords:** COVID-19, anxiety, depression, healthcare worker, general public

## Abstract

**Background:** Mental health burden has been massively reported during the COVID-19 pandemic period. Aiming to summarise these data, we present a meta-review of meta-analyses that evaluated the impact of COVID-19 pandemic on anxiety, depressive and stress symptoms, psychological distress, post-traumatic stress disorder/symptoms (PTSD), and sleep disturbance, reporting its prevalence on general public (GP) and health care workers (HCW).

**Methods:** A search was performed in the PubMed, EMBASE, and the Web of Science. Sleep disturbances, psychological distress, stress, and burnout were grouped as “Psychophysiological stress,” and anxiety, depression, and PTSD were grouped as “Psychopathology.” A random-effects model, calculating the pooled prevalence together with 95% confidence interval was performed for each domain. Subgroup analyses were performed for each population type (GP and HCW) and for each mental health outcome. For anxiety and depression, subgroup analysis for population type was performed. Heterogeneity is reported as *I*^2^. Publication bias was assessed through visual inspection of the funnel plot, and further tested by Egger's test and trim and fill analyses.

**Results:** A total of 18 meta-analyses were included. The prevalence of psychophysiological stress was 31.99% (CI: 26.88–37.58, *I*^2^ = 99.9%). HCW showed a higher prevalence (37.74%, CI: 33.26–42.45, *I*^2^ = 99.7%) than the GP (20.67%, 15.07–27.66, *I*^2^ = 99.9%). The overall prevalence of insomnia, psychological distress, and stress were, respectively, 32.34% (CI: 25.65–39.84), 28.25% (CI: 18.12–41.20), and 36% (CI: 29.31–43.54). Psychopathology was present at 26.45% (CI: 24.22–28.79, *I*^2^ = 99.9%) of the sample, with similar estimates for population (HCW 26.14%, CI: 23.37–29.12, *I*^2^ = 99.9%; GP: 26.99%, CI: 23.41–30.9, *I*^2^ = 99.9%). The prevalence of anxiety, depression, and PTSD was 27.77% (CI: 24.47–31.32), 26.93% (CI: 23.92–30.17), and 20% (CI: 15.54–24.37), respectively. Similar proportions between populations were found for anxiety (HCW = 27.5%, CI: 23.78–31.55; GP = 28.33%, CI: 22.1–35.5) and depression (HCW = 27.05%, CI: 23.14–31.36; GP = 26.7%, CI: 22.32–31.59). Asymmetry in the funnel plot was found, and a slight increase in the estimate of overall psychopathology (29.08%, CI: 26.42–31.89) was found after the trim and fill analysis.

**Conclusions:** The prevalence of mental health problems ranged from 20 to 36%. HCW presented a higher prevalence of psychophysiological stress than the general population.

**Systematic Review Registration:**https://www.crd.york.ac.uk/PROSPERO/display_record.php?RecordID=252221, identifier: CRD42021252221.

## Introduction

On March 11, 2020, the World Health Organisation (WHO) declared the new coronavirus disease (COVID-19) a pandemic (World Health Organization, 2020). The pandemic began in December, 2019, in Wuhan, China, and spread all over the world. The new coronavirus identified as SARS-CoV-2 has infected 206,958,371 people and caused 4,357,179 deaths to date **(**August 16, 2021 [12:23pm CEST]) (World Health Organization, 2019).

Among the procedures to prevent dissemination of the virus, social distancing and quarantining have been advised by authorities (World Health Organization, 2020). It is important to mention that the social isolation and lockdown brought important economic consequences, especially for self-employed workers. Moreover, the fear of contamination also presents an important negative impact on mental health, such as increased depressive and anxious symptoms, worsening cognitive performance and disrupting sleep (Brooks et al., 2020; Ornell et al., 2020).

Although studies during COVID-19 are mostly based on online surveys, using self-reported questionnaires applied *via* web, evidence from previous and recent work shows that the overall prevalence of psychopathology symptoms of depression and anxiety since the onset of COVID-19 was 31.5 and 31.9%, respectively (Wu et al., 2021). For COVID-19 patients, the prevalence of depression was 41.7% and for anxiety 42.3% (Wu et al., 2021). According to WHO in 2017, the depression rate among the global population was 4.4% and 3.6% for anxiety disorders (World Health Organization, 2017). These results, besides the bias towards region and methodological issues, suggest a huge impact of the COVID-19 pandemic on the psychological wellbeing not only to the general public (GP) but especially for health workers due the high demand and extenuating working hours (Luo et al., 2020; Li et al., 2021; Wu et al., 2021). In fact, data in a recent study showed the prevalence of anxiety in health care workers (HCW) at 25%, with a highlight to the frontline HCW with 43% (Santabárbara et al., 2021a,b).

Despite the mental health of all people being impacted, those with previous diagnoses or symptoms of mental disorders and impaired cognition require special attention in quarantine and social isolation. Once they might face additional difficulties to follow recommendations and to understand the limitations and may also face limited mental health assistance (Ornell et al., 2020). During the COVID-19 pandemic, families are even more challenged to lead their lives with people with mental disorders confined at home (Ornell et al., 2020).

With the confinement and social isolation along with eventual economic, health, and political crises, different populations are under a lot of stress due to the increase in the fear of contamination, the burden, and the intensity of work for those who stand at the frontline such as HCW (Santabárbara et al., 2021a,b). In addition, all groups of people are subject to experience loneliness, fear of staying away from the family (Schellekens and van der Lee, 2020), anxiety (Schuch et al., 2020), depression (Schuch et al., 2020), stress (Burtscher et al., 2020), insomnia/sleep disorders (Partinen, 2021), and psychological stress (Li et al., 2020).

Meta-reviews are useful to provide an integrated view of the several studies that are currently being conducted regarding COVID-19. Recently, an umbrella review assessed seven meta-analytic studies published between 2019 and 2020, revealing a similar estimated prevalence of anxiety (24.94%) and depressive (24.83%) symptoms in HCW (Sahebi et al., 2021). However, estimates for the GP as well as the comparison between these two populations are lacking. Therefore, an updated meta-review addressing these issues would benefit the literature providing a framework for the impact of the ongoing pandemic on the mental health of the public in general and health workers.

Based on these assumptions we proposed a meta-review to (i) aggregate and evaluate the top-tier evidence for situational analysis of the present scenario, collecting evidence of meta-analyses currently available from several countries, and (ii) quantify the prevalence of various psychological morbidities among the general population and health care professionals in the midst of the COVID-19 pandemic. To achieve this, we identified, synthesised, and appraised available data from meta-analyses examining the mental health outcomes during the COVID-19 pandemic.

## Materials and Methods

This systematic review and meta-analysis were conducted in accordance with the recommendations outlined in the Preferred Reporting Items for Systematic Reviews and Meta-Analyses (PRISMA) statement (Moher et al., 2009). The review protocol was registered at PROSPERO as CRD42021252221.

### Search Strategy and Study Selection

A search from 2019 up to March 2, 2021, was carried out, according to the PO (population: GP and HCW; outcome: prevalence/proportion of depression, anxiety, stress, or sleep disorders) framework, and using the following electronic databases: PubMed, Embase, and Web of Science. The search strategy used in PubMed combined the terms “coronavirus” or “SARS-COV-2” or “COVID-19,” and “anxiety” or “mental health” or “psychological” or “humor” or “mood” or “affective symptoms” or “mood states” or “depressive symptoms” or “depression” or “affective disorders.” The searches for other databases were slightly adapted (Supplementary Table 1). Filters of date of publication (2019–2021) and study type (meta-analysis) were applied when available. Titles and abstracts of retrieved articles were individually evaluated by two reviewers (GMSJ and MLPMG) to assess their eligibility for meta-review. Study inclusions were checked by a third reviewer (VT). Study abstracts that did not provide sufficient information according to the inclusion criteria were retrieved for full-text evaluation. A search on Google Scholar and in the references of included studies was further performed to identify any non-included relevant study.

### Eligibility Criteria

Articles were eligible for inclusion if they met the following criteria: (1) consisted of meta-analytical study assessing symptoms of depression, anxiety, and stress, or sleep disorders, assessed by validated screening instruments; (2) was assessed in GP or HCW; (3) peer-reviewed articles published in English; (3) adult participants (≥18 years of age); (4) provided sufficient information to calculate the prevalence/proportion of symptoms of depression, anxiety, stress, or sleep disorders among HCW and GP excluding COVID patients (e.g., percentage or sample size and number of events). Articles were excluded if (1) consisted of systematic review or other type excluding meta-analysis; (2) did not present prevalence as the effect size; (3) assessed outcomes only in patients; or (4) full-text was unavailable.

### Data Extraction

Data were blindly extracted by two reviewers (GMSJ and MLPMG) and compiled into an Excel spreadsheet. Relevant data were collected regarding study characteristics (outcome, population type, number of studies, and sample size by outcome and population type) and study results (pooled outcome prevalence by population and *I*^2^).

### Statistical Analysis

The analyses were conducted using the *meta* package of R software (version 4.0.3). The effect size was the prevalence rate. Between-study variability was examined for heterogeneity, using the *I*^2^ statistic for quantifying inconsistency (Higgins et al., 2003). Heterogeneity thresholds were set at *I*^2^ = 25% (low), *I*^2^ = 50% (moderate), and *I*^2^ = 75% (high) (Higgins et al., 2003). A random-effects model was applied to pool the data for each analysis. For adequate statistical power, a minimum of five studies were included in the pooled random-effects analysis (Jackson and Turner, 2017). Subgroup analysis for population type (GP and HCW) was performed for anxiety and depression, since the number of studies for each population was ≥5. Cochran's *Q* and degrees of freedom were reported for comparison tests between subgroups as *Q*(*df*). The level of significance was set at *p* ≤ 0.05 for subgroup comparisons. Publication bias was assessed using funnel plots, and Egger's test of effect size against its standard error, when *k* ≥ 10.

## Results

### Study Characteristics

**Figure 1:**
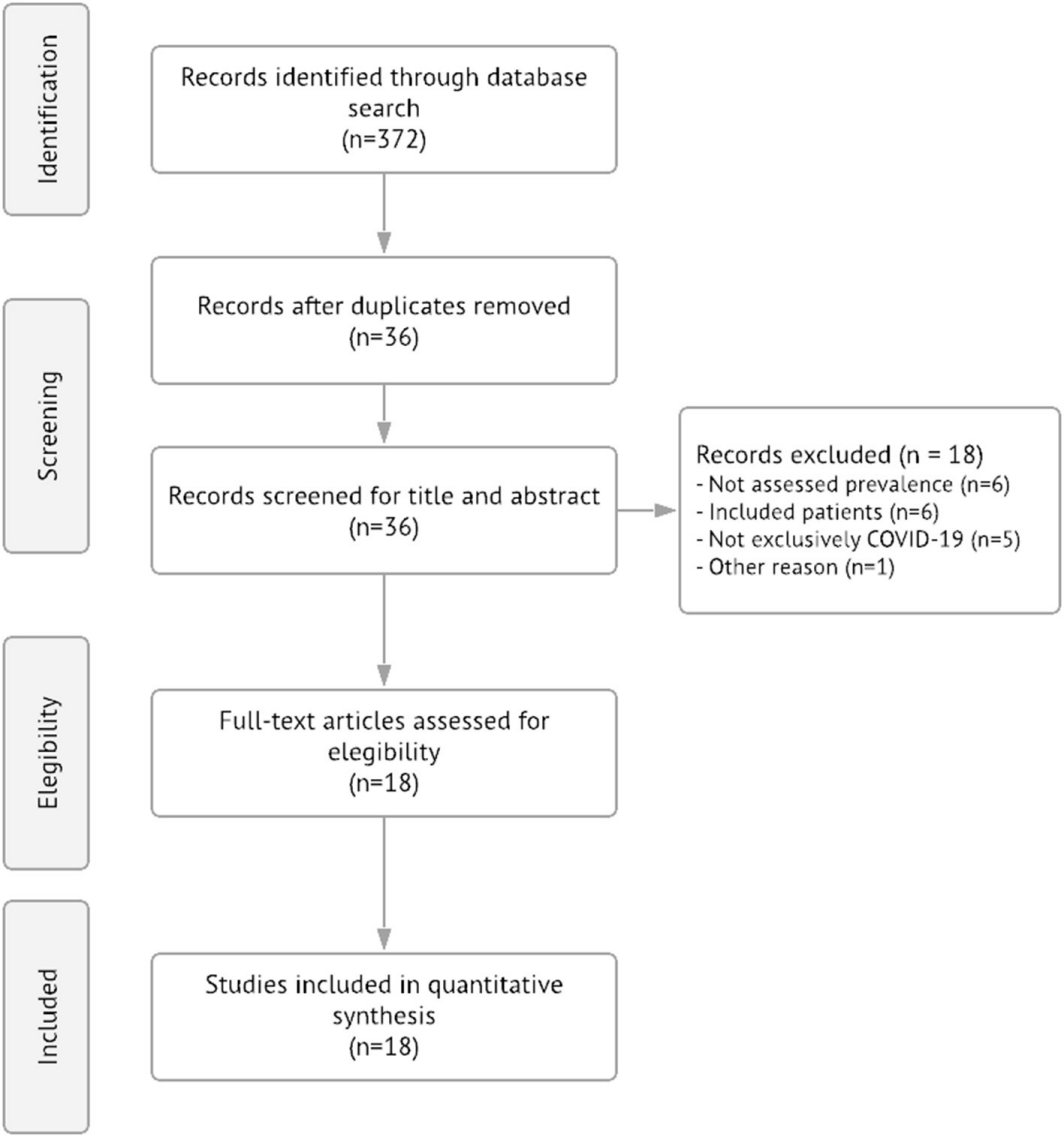
PRISMA diagram summarizing the records retrieval and workflow.

A total of 372 studies were retrieved (156 from PubMed, 51 from Embase and 165 from Web of Science) and 36 were selected after removing duplicates. After the title/abstract screening, 18 meta-analyses (*n* = 1,074,438) were found to be eligible for analysis (Figure 1). The majority of articles included studies performed in Asian countries (*k* = 17, 94.4%), followed by European countries (*k* = 10, 55.6%), South and Central Americas (*k* = 6, 33.3%) and North America (*k* = 6, 33.3%) with the same proportion, Africa countries were included in 5 articles (27.1%), and finally Oceania countries (*k* = 2, 11.1%). One article (5.6%) did not have the information about the countries of the analyzed studies (Supplementary Table 2). Information regarding the quality assessment of included meta-analyses can be found in Supplementary Table 2.

Stress was assessed by five studies (Batra et al., 2020; Cooke et al., 2020; Salari et al., 2020a,c; Al Maqbali et al., 2021), three among HCW (Batra et al., 2020; Salari et al., 2020c; Al Maqbali et al., 2021), and two among the GP (Cooke et al., 2020; Salari et al., 2020a). Distress was assessed in GP and HCW by one study (Wu et al., 2021); and psychological distress was assessed by two others, one in GP and HCW subjects (Cénat et al., 2021) and the another in HCW only (Batra et al., 2020). Sleep disturbance was assessed by two studies, one in HCW (Al Maqbali et al., 2021) and another in physicians and nurses (Salari et al., 2020b); while four studies assessed insomnia (Batra et al., 2020; Pappa et al., 2020; Cénat et al., 2021; Wu et al., 2021), two of them in HCW and GP (Cénat et al., 2021; Wu et al., 2021) and the other two in HCW only (Batra et al., 2020; Cénat et al., 2021). One study assessed burnout in HCW (Batra et al., 2020) (Supplementary Table 2).

Anxiety was assessed in 16 studies (Bareeqa et al., 2020; Batra et al., 2020; Lasheras et al., 2020; Luo et al., 2020; Panda et al., 2020; Pappa et al., 2020; Salari et al., 2020a,b,c; Al Maqbali et al., 2021; Cénat et al., 2021; da Silva and Neto, 2021; Deng et al., 2021; Li et al., 2021; Santabárbara et al., 2021b; Wu et al., 2021), 8 among GP (Lasheras et al., 2020; Luo et al., 2020; Panda et al., 2020; Salari et al., 2020a; Cénat et al., 2021; Deng et al., 2021; Santabárbara et al., 2021b; Wu et al., 2021), and 15 among HCW (Bareeqa et al., 2020; Batra et al., 2020; Luo et al., 2020; Pappa et al., 2020; Salari et al., 2020c; Al Maqbali et al., 2021; Cénat et al., 2021; da Silva and Neto, 2021; Deng et al., 2021; Li et al., 2021; Santabárbara et al., 2021a; Wu et al., 2021) (Supplementary Table 2).

Depression was assessed by 13 studies (Bareeqa et al., 2020; Batra et al., 2020; Luo et al., 2020; Panda et al., 2020; Pappa et al., 2020; Salari et al., 2020a,c; Al Maqbali et al., 2021; Cénat et al., 2021; da Silva and Neto, 2021; Deng et al., 2021; Li et al., 2021; Wu et al., 2021), 4 assessed in GP and HCW (Luo et al., 2020; Cénat et al., 2021; Deng et al., 2021; Wu et al., 2021), 2 in GP only (Panda et al., 2020; Salari et al., 2020a), and 7 in HCW only (Bareeqa et al., 2020; Batra et al., 2020; Pappa et al., 2020; Salari et al., 2020c; Al Maqbali et al., 2021; da Silva and Neto, 2021; Li et al., 2021) (Supplementary Table 2).

Four studies assessed the post-traumatic stress disorder/symptoms (PTSD) (Batra et al., 2020; Cooke et al., 2020; Cénat et al., 2021; Li et al., 2021), 2 of them in HCW (Batra et al., 2020; Li et al., 2021), 1 in GP (Cooke et al., 2020), and 1 in both GP and HCW subjects (Cénat et al., 2021) (Supplementary Table 2).

For the analysis, we merged “Distress” and “Burnout” in “Psychological distress,” and “Sleep disturbance” with “Insomnia” and then this groups was named as “Insomnia.” Then, the outcomes “Stress,” “Psychological distress,” and “Insomnia” were pooled into the so-called “Psychophysiological stress” domain, in order to get the overall estimate of stress-related outcomes. Similarly, “Anxiety,” “Depression,” and “PTSD” were pooled into the “Psychopathology” domain.

### Pooled Estimates for Psychophysiological Stress

**Figure 2:**
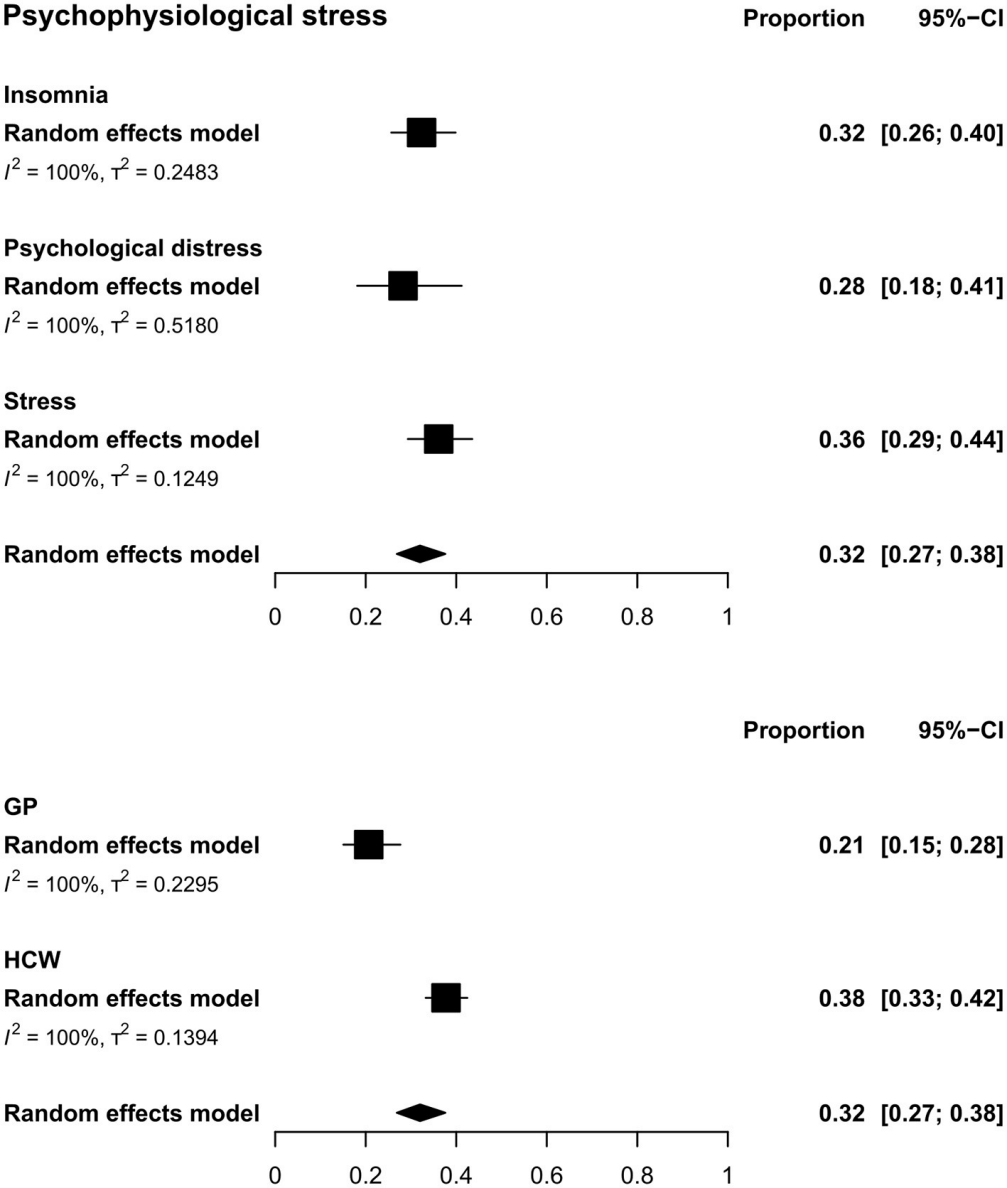
Pooled prevalence for psychophysiological stress by outcomes (upper) and by population type (lower). In the upper panel, squares represent the overall estimate prevalence for each outcome without specifying the population. In the lower panel, squares represent the overall estimate prevalence for each population without specifying the outcome. In both cases, diamonds represent the overall prevalence for the psychophysiological stress domain. GP, general public; HCW, health care workers.

The overall estimated prevalence for psychophysiological stress was 31.99% (CI: 26.88–37.58, τ^2^ = 0.32, *I*^2^ = 99.9%) (Figure 2). The prevalence between population type was significantly different [*Q*(1) = 14.76; *p* = 0.0001], where HCW showed a higher prevalence (37.74%, CI: 33.26–42.45, τ^2^ = 0.14, *I*^2^ = 99.7%) than the GP (20.67%, CI: 15.07–27.66, τ^2^ = 0.23, *I*^2^ = 99.9%) (Figure 2).

For GP and HCW the overall prevalence of stress was 36.12% (CI: 29.31–43.54, τ^2^ = 0.12, *I*^2^ = 99.7%). Whereas for psychological distress, a prevalence of 28.25 (CI: 18.12–41.20, τ^2^ = 0.52, *I*^2^ = 99.9%) was found and for insomnia it was 32.34 (CI: 25.65–39.84, τ^2^ = 0.25, *I*^2^ = 99.8%) (Figure 2, Supplementary Figure 1).

### Pooled Estimates for Psychopathology

**Figure 3:**
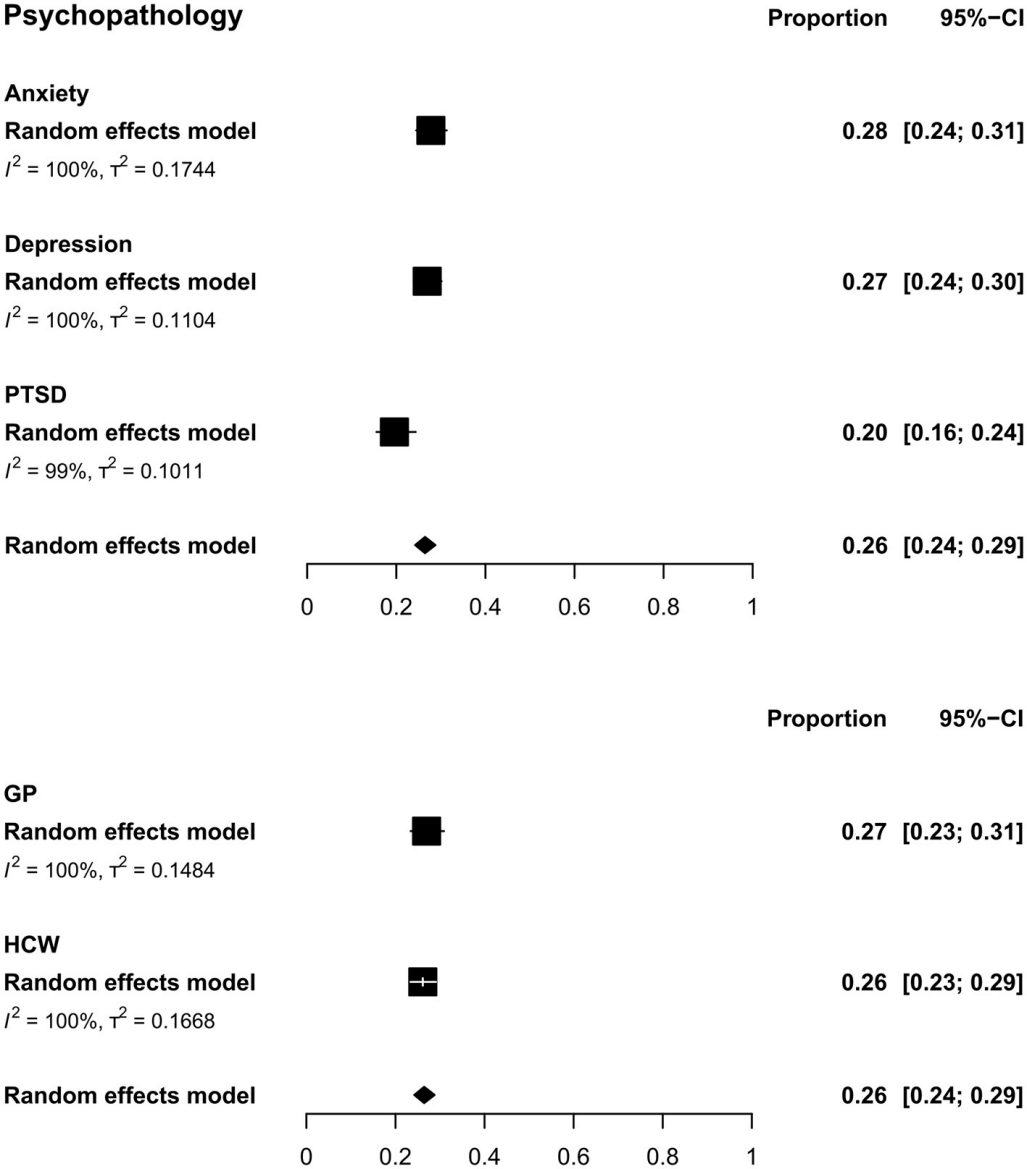
Pooled prevalence for psychopathology by outcomes (upper) and by population type (lower). In the upper panel, squares represent the overall estimate prevalence for each outcome without specifying the population. In the lower panel, squares represent the overall estimate prevalence for each population without specifying the outcome. In both cases, diamonds represent the overall prevalence for the psychopathology domain. PTSD, post-traumatic stress disorder; GP, general public; HCW, health care workers.

The overall estimated prevalence for psychopathology was 26.45% (CI: 24.22–28.79, τ^2^ = 0.16, *I*^2^ = 99.9%) (Figure 3). The was no difference [*Q*(1) = 0.12; *p* = 0.724] between the prevalence of psychopathology in HCW (26.14%, CI: 23.37–29.12, τ^2^ = 0.17, *I*^2^ = 99.9%) and in the GP (26.99%, CI: 23.41–30.9, τ^2^ = 0.15, *I*^2^ = 99.9%) (Figure 3).

#### Anxiety

The overall prevalence of anxiety was 27.77% (CI: 24.47–31.32; τ^2^ = 0.17, *I*^2^ = 99.9%) (Figure 3). No difference was found for between-population analysis under the random effects model analysis [*Q*(1) = 0.04, *p* = 0.83]. For GP, the pooled prevalence was 28.33% (CI: 22.1–35.5; τ^2^ = 0.23, *I*^2^ = 100.0%). For HCW, the prevalence was 27.5% (CI: 23.78–31.55; τ^2^ = 0.15, *I*^2^ = 99.9%) (Supplementary Figure 2).

#### Depression

The overall prevalence of depression was 26.93% (CI: 23.92–30.17; τ^2^ = 0.11, *I*^2^ = 99.9%) (Figure 3). No difference between populations was found under the random effects model analysis [*Q*(1) = 0.01, *p* = 0.91]. For GP, the pooled prevalence was 26.7% (CI: 22.32–31.59; τ^2^ = 0.08, *I*^2^ = 99.8%). For HCW, a prevalence of 27.05% was found (CI: 23.14–31.36; τ^2^ = 0.12, *I*^2^ = 99.9%) (Supplementary Figure 2).

#### Post-traumatic Stress Disorder/Symptoms

The overall prevalence of PTSD was 19.58% (CI: 15.54–24.37, τ^2^ = 0.10, *I*^2^ = 99.5%) (Figure 3). Due to the small number of studies by populations, no between-population subgroup analysis was performed for PTSD.

### Publication Bias

Visual inspection of funnel plots suggests bias for psychophysiological stress, psychopathology, anxiety, and depression (Supplementary Figure 3). Asymmetry in the funnel plots was confirmed by the Egger's test (psychophysiological stress: *t*_(23)_ = −0.01, *p* = 0.99; psychopathology: *t*_(50)_ = −0.67, *p* = 0.50; anxiety: *t*_(21)_ = −0.24, *p* = 0.81; depression: *t*_(15)_ = −0.56, *p* = 0.58). The trim and fill analysis adjusted estimates for psychophysiological stress to 31.99% (CI: 26.88–37.58), psychopathology to 29.08% (CI: 26.42–31.89), anxiety to 27.77 (CI: 24.47–31.33), and depression to 26.94% (23.93–30.17). The virtual lack of conspicuous change in psychophysiological stress, anxiety, and depression may be due to the high between-study heterogeneity.

## Discussion

In this meta-review, we pooled data from 18 meta-analyses evaluating the prevalence of general psychophysiological stress and psychopathology among the GP and HCW populations during the COVID-19 pandemic. The majority of meta-analyses included studies performed in Asian countries.

We found an overall prevalence of 32% of psychophysiological stress, representing 32% of insomnia/sleep disturbance, 28% of psychological stress, and 36% of stress. The prevalence of psychophysiological stress was higher for HCW (38%) than for the GP (21%). However, psychophysiological stress issues are often reported for HCW even in the absence of disease outbreaks (Liu et al., 2019; Lee et al., 2020; Woo et al., 2020), so these results should be interpreted with some caution.

Regarding psychopathology, an overall prevalence of 26% was found, with a similar prevalence for anxiety (28%), and depression (27%), and 20% of PTSD. A similar prevalence of psychopathology was observed in the HCW (26%) and in the GP (27%). A subgroup analysis by population for anxiety and depression showed similar prevalence for HCW (anxiety: 27.5%, depression: 27.05%) and the GP (anxiety: 28.33%, depression: 26.7%).

A previous review of meta-analyses found slightly lower estimates for anxiety (24.94%) and depression (24.83%) in HCW during the COVID-19 pandemic (Sahebi et al., 2021) as compared to our findings. The review included seven studies published between January and October 2020. Therefore, since our study included studies also published in 2021, with a total of 18 studies published between May 2020 and March 2021, the difference in the estimates could be due to this temporal lag and may suggest an increase in the prevalence of these outcomes in this population.

When compared with the estimates of previous viral epidemic outbreaks, for instance, Severe Acute Respiratory Syndrome (SARS), Middle Eastern respiratory syndrome (MERS), H1N1, in HCW, Serrano-Rippol and colleagues found a lower proportion for depression (24%) and a higher proportion for anxiety (30%) (Serrano-Ripoll et al., 2020). The prevalence of PTSD (13%) was lower for HCW than our overall estimate (not specifying population) (Serrano-Ripoll et al., 2020). It is important to highlight that these previous estimates were made by pooling several diseases within the time range of 2002–2020. Since our estimates bring homogeneous data regarding COVID-19, we may speculate that in this 2019–2021 timeframe, COVID-19 only reaches similar levels of anxiety and surpasses depression and PTSD rates of these past viral outbreaks together.

It was shown that lockdown has a small but significant and heterogeneous effect on depression and anxiety (Prati and Mancini, 2021). Therefore, possible solutions to help coping these adversities during the social isolation and the frontline care are needed. Cabarkapa and colleagues point some ways to deal with psychological risks in HCW, such as self-coping strategies, psychoeducation, and awareness in the workplace (Cabarkapa et al., 2020). Complementary therapies, such as nutraceuticals and lifestyle changes are suggested as a way to reduce COVID-19-induced inflammation overload, once it would help to reduce negative mental health symptoms (Sarris et al., 2014, 2021; Neto et al., 2020), and improve sleep even in COVID-19 patients (Ding et al., 2021).

In addition to those approaches, we also encourage the use of feasible individual homemade practices to address such issues. For instance, physical exercise is related to physical, psychological, and cognitive improvements in mood and general health (Schuch et al., 2016; Ashdown-Franks et al., 2019; Wolf et al., 2021). Mind–body integrative practices such as mindfulness meditation and yoga have also shown to be effective in reducing psychophysiological distress while improving positive psychological measures (Cahn et al., 2017; Pascoe et al., 2017; Goldberg et al., 2018; Solhaug et al., 2019; Sousa et al., 2021). In addition, cultivating mind–body practices flourishes positive feelings about the self and toward others, such as (self-)compassion, empathy, and pro-sociality (Garland et al., 2015; Voci et al., 2019), what may be useful to face social distancing in a less detrimental way.

This study has some key limitations, such as the high heterogeneity and the publication bias. In addition, it should be noted that the studies comprising the present meta-review were conducted when there were no wide-ranging vaccines or variants of concern. Nevertheless, our study provides a current overview of the burden of COVID-19 in the GP and in HCW. Having these measures is crucial for the development and proper direction of public policies and government campaigns in order to mitigate the worsening of this scenario as well as for paving the way to face similar future events.

In summary, in this study, we showed, by the overall pooling of other meta-analytical reports regarding COVID-19 burden of emotional outcomes, high proportions of psychophysiological stress in the general population and in HCW, and higher prevalence of psychopathology in HCW compared with the GP.

## Data Availability Statement

The original contributions presented in the study are included in the article/Supplementary Material, further inquiries can be directed to the corresponding author.

## Author Contributions

VT performed the searches and inspected selection process. GS and MG selected studies and extracted data. GS, VT, NG-C, GL-A, FS, MG, and MC drafted and edited the manuscript. All authors contributed to the article and approved the submitted version.

## Funding

NG-C is supported by National Science and Technology Institute for Translational Medicine (INCT-TM Fapesp 2014/50891-1; CNPq 465458/2014-9). GS is supported by Coordination for the Improvement of Higher Education Personnel (CAPES, Proc. No. 88887.597821/2021-00). The funders had no role in study conception, data extraction and analysis, decision to publish, or in the preparation of the manuscript.

## Conflict of Interest

The authors declare that the research was conducted in the absence of any commercial or financial relationships that could be construed as a potential conflict of interest.

## Publisher's Note

All claims expressed in this article are solely those of the authors and do not necessarily represent those of their affiliated organizations, or those of the publisher, the editors and the reviewers. Any product that may be evaluated in this article, or claim that may be made by its manufacturer, is not guaranteed or endorsed by the publisher.
