# Supplementary material for: Mental Health in COVID-19 Pandemic: A Meta-Review of Prevalence Meta-Analyses
Source: Front Psychol. 2021 Sep 21;12:703838. doi: 10.3389/fpsyg.2021.703838 (PMC8490780; doi:10.3389/fpsyg.2021.703838)
Supplement: Supplementary file 1 [file Table_1.PDF]

Search strategies for each database

Database	Search strategy
PubMed	((coronavirus) OR (SARS-COV-2) OR (COVID-19)) AND ((anxiety) OR ("mental health") OR (psychological) OR (humor) OR (mood) OR ("affective symptoms") OR ("mood states") OR ("depressive symptoms") OR (depression) OR ("affective disorders"))
Embase	(coronavirus OR SARS-COV-2 OR COVID-19) AND (anxiety OR "mental health" OR psychological OR humor OR mood OR "affective symptoms" OR "mood states" OR "depressive symptoms" OR depression OR "affective disorders")
Web of Science	((coronavirus OR SARS-COV-2 OR COVID-19) AND (anxiety OR "mental health" OR psychological OR humor OR mood OR "affective symptoms" OR "mood states" OR "depressive symptoms" OR depression OR "affective disorders"))
