# Supplementary material for: Mental Health in COVID-19 Pandemic: A Meta-Review of Prevalence Meta-Analyses
Source: Front Psychol. 2021 Sep 21;12:703838. doi: 10.3389/fpsyg.2021.703838 (PMC8490780; doi:10.3389/fpsyg.2021.703838)
Supplement: Supplementary file 2 [file Table_2.PDF]

Study	Outcome	HCW			GP			Mean quality [max score]	Tool	Continent
		k (n)	Prevalence	I^2	k (n)	Prevalence	I^2			
Bareeqa et al. (2020)	Depression	8 (10267)	31.5	-	-	-	-	3.37 [6]	Modified NOS	AS
	Anxiety	8 (10267)	23.7	-	-	-	-			
Batra et al. (2020)	Anxiety	46 (51596)	34.4	99.1	-	-	-	7.21 [9]	NIH quality assessment tool	AS, EU, SA, NA
	Depression	46, (53164)	31.8	99.2	-	-	-			
	Stress	17 (16235)	40.3	99.1	-	-	-			
	PTSS	6 (3676)	11.4	99.2	-	-	-			
	Insomnia	11 (18546)	27.8	98.1	-	-	-			
	Psychological distress	12 (30963)	46.1	99.6	-	-	-			
	Burnout	3 (2487)	37.4	98.6	-	-	-			
Cénat et al. (2021)	Depression	18 (37076)	13.75	-	28 (71317)	17.05	-	8.25 [9]	JBI	AS, EU, SA, NA
	Anxiety	23 (37076)	15.86	-	31 (84297)	14.62	-			
	Insomnia	6 (7379)	36.52	-	8 (34790)	16.45	-			
	PTSD	4 (4196)	20.91	-	9 (26253)	22.43	-			
	Psychological distress	9 (8487)	16.88	-	10 (59668)	10.19	-			
Cooke et al. (2020)	PTSD	-	-	-	8 (9232)	23.88	99.99	3.07 [-]	Not informed	AS, EU, NA, AF
	Stress	-	-	-	8 (14323)	24.84	99.997			
Da Silva et al. (2021)	Depression	7 (7102)	33	-	-	-	-	Not informed	-	AS
	Anxiety	7 (7102)	36	-	-	-	-			
Deng et al. (2021)	Depression	20 (11438)	31	98.4	10 (16094)	26	99.5	7.32 [11]	AHRQ's checklist	Not informed
	Anxiety	22 (11401)	40	98.6	10 (16386)	22	99.3			
Lasheras et al. (2020)	Anxiety	-	-	-	8 (11710)	28	97.51	7.63 [9]	JBI	AS, SA
Li et al. (2021)	Depression	55 (84666)	21.7	99.3	-	-	-	Not applied	Agarwal et al.	AS, EU, NA

	Anxiety	57 (74280)	22.1	99.4	-	-	-			
	PTSD	9 (24439)	21.5	99.7	-	-	-			
Luo et al. (2020)	Anxiety	13 (14357)	26	98.94	24 (67292)	32	99.78	11.29 [12]	McMaster University critical appraisal tool	AS, EU, SA
	Depression	14 (11819)	25	99.19	14 (60353)	27	99.7			
Maqbali et al. (2021)	Stress	40 (27034)	43	98	-	-	-	5.91 [9]	NSO	AS, EU, NA, AF
	Anxiety	73 (81561)	37	99	-	-	-			
	Depression	62 (76992)	35	99	-	-	-			
	Sleep disturbance	18 (10697)	43	97	-	-	-			
Panda et al. (2020)	Anxiety	-	-	-	3 (257)	52	61	3 fair quality	ROBINS-I	AS
	Depression	-	-	-	3 (257)	27.4	34			
Pappa et al. (2020)	Anxiety	12 (27756)	23.2	99	-	-	-	3.15 [5]	Modified NSO	AS
	Depression	10 (31014)	22.8	99.62	-	-	-			
	Insomnia	5 (8558)	34.32	98	-	-	-			
Salari et al. (2020a)	Stress	-	-	-	5 (9074)	29.6	96.8	All scored ≥ 16 [32]	STROBE checklists	AS, EU, AF
	Anxiety	-	-	-	17 (63439)	31.9	99.3			
	Depression	-	-	-	14 (44531)	33.7	99.4			
Salari et al. (2020b)	Sleep disturbance*	5 (2123)	34.8	97.4	-	-	-	All scored ≥ 16 [32]	STROBE checklists	AS
	Sleep disturbance**	6 (3745)	41.6	97.3	-	-	-			
Salari et al. (2020c)	Depression	21 (-)	24.3	98.9	-	-	-	All scored ≥ 16 [32]	STROBE checklists	AS, EU, OC
	Anxiety	23 (19768)	25.8	98.5	-	-	-			
	Stress	9 (3719)	45	99.1	-	-	-			
Santabárbara et al. (2021a)	Anxiety***	59 (58565)	25	99.12	-	-	-	7.08 [10]	JBI	AS, EU, SA, OC, AF
	Anxiety**	17 (6875)	27	97.8	-	-	-			
	Anxiety*	13 (5177)	17	95.55	-	-	-			

	Anxiety****	13 (5880)	43	99.5	-	-	-			
Santabárbara et al. (2021b)	Anxiety	-	-	-	43 (161556)	25	99.7	7.30 [9]	JBI	AS, EU, SA, NA, AF
Wu et al. (2021)	Depression	23 (41889)	31	99.5	17 (69697)	31.5	99.8	Not applied	STROBE	AS
	Anxiety	23 (50143)	29	99.4	18 (57898)	29.8	99.8			
	Distress	5 (10165)	41.2	99.8	4 (56528)	31.1	97.2			
	Insomnia	7 (13375)	47.3	98.7	1 (7238)	18.2	-			

* Physicians/Medical Doctors, ** Nurses, ***HCW, ****Frontline HCW; AS = Asia, EU = Europe, SA = South and Central Americas, NA = North America, OC = Oceania, AF = Africa. AHRQ = Agency for Healthcare Research and Quality; JBI = Joanna Briggs Institute; NIH = ; NOS = Newcastle-Ottawa Scale; ROBINS-I = Risk Of Bias In Non-randomised Studies - of Interventions; STROBE = Strengthening the Reporting of Observational Studies in Epidemiology.
