# Supplementary material for: Mental Health in COVID-19 Pandemic: A Meta-Review of Prevalence Meta-Analyses
Source: Front Psychol. 2021 Sep 21;12:703838. doi: 10.3389/fpsyg.2021.703838 (PMC8490780; doi:10.3389/fpsyg.2021.703838)
Supplement: Supplementary file 3 [file Image_1.PDF]

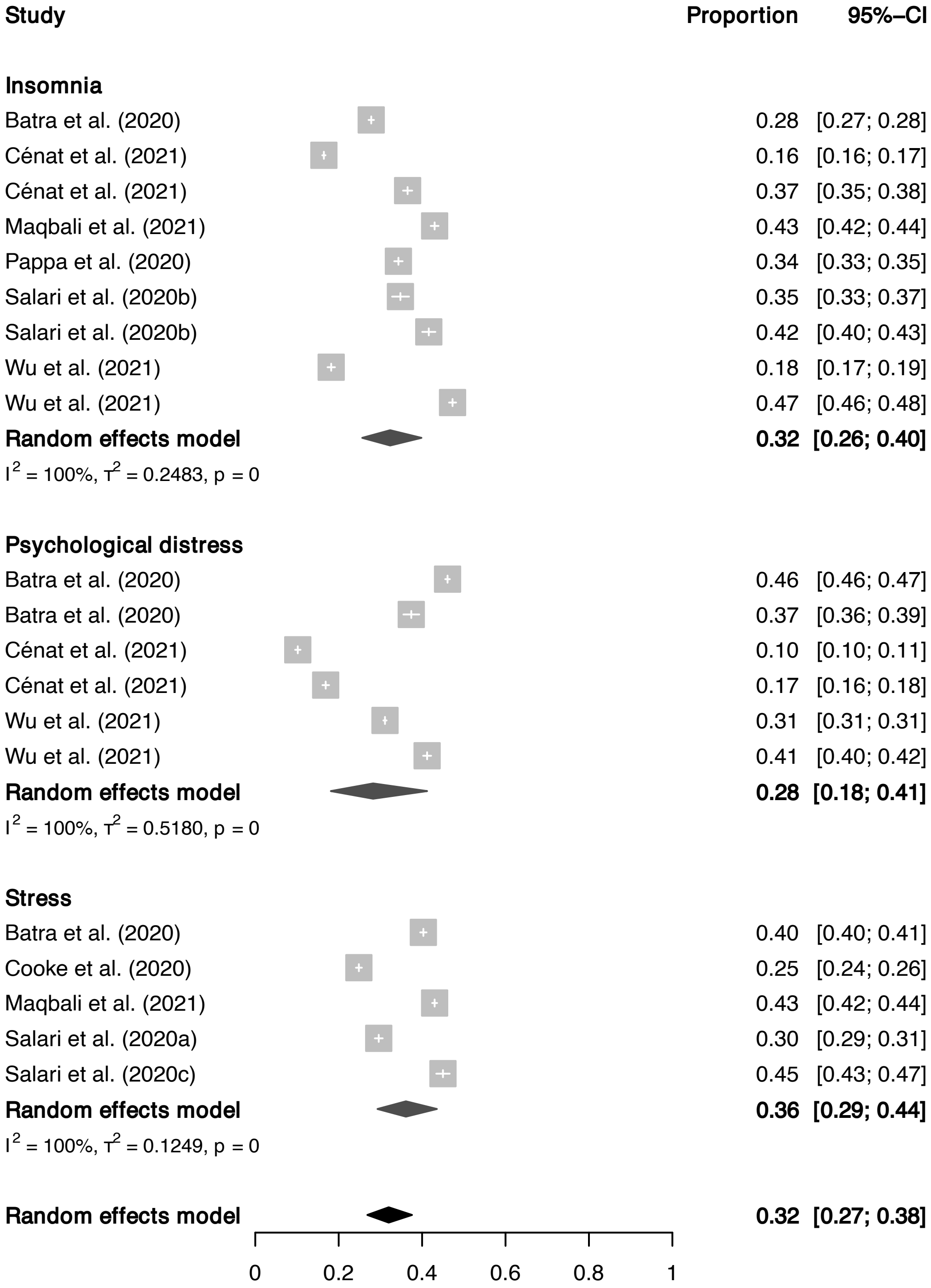
Study
Proportion
95%–CI
Insomnia
Batra et al. (2020) 0.28 [0.27; 0.28]
Cénat et al. (2021) 0.16 [0.16; 0.17]
Cénat et al. (2021) 0.37 [0.35; 0.38]
Maqbali et al. (2021) 0.43 [0.42; 0.44]
Pappa et al. (2020) 0.34 [0.33; 0.35]
Salari et al. (2020b) 0.35 [0.33; 0.37]
Salari et al. (2020b) 0.42 [0.40; 0.43]
Wu et al. (2021) 0.18 [0.17; 0.19]
Wu et al. (2021) 0.47 [0.46; 0.48]
Random effects model 0.32 [0.26; 0.40]
$I^2 = 100\%, \tau^2 = 0.2483, p = 0$
Psychological distress
Batra et al. (2020) 0.46 [0.46; 0.47]
Batra et al. (2020) 0.37 [0.36; 0.39]
Cénat et al. (2021) 0.10 [0.10; 0.11]
Cénat et al. (2021) 0.17 [0.16; 0.18]
Wu et al. (2021) 0.31 [0.31; 0.31]
Wu et al. (2021) 0.41 [0.40; 0.42]
Random effects model 0.28 [0.18; 0.41]
$I^2 = 100\%, \tau^2 = 0.5180, p = 0$
Stress
Batra et al. (2020) 0.40 [0.40; 0.41]
Cooke et al. (2020) 0.25 [0.24; 0.26]
Maqbali et al. (2021) 0.43 [0.42; 0.44]
Salari et al. (2020a) 0.30 [0.29; 0.31]
Salari et al. (2020c) 0.45 [0.43; 0.47]
Random effects model 0.36 [0.29; 0.44]
$I^2 = 100\%, \tau^2 = 0.1249, p = 0$
Random effects model 0.32 [0.27; 0.38]
0
0.2
0.4
0.6
0.8
1
