# Supplementary figures and images for: Mental Health in COVID-19 Pandemic: A Meta-Review of Prevalence Meta-Analyses
Source: Front Psychol. 2021 Sep 21;12:703838. doi: 10.3389/fpsyg.2021.703838 (PMC8490780; doi:10.3389/fpsyg.2021.703838)

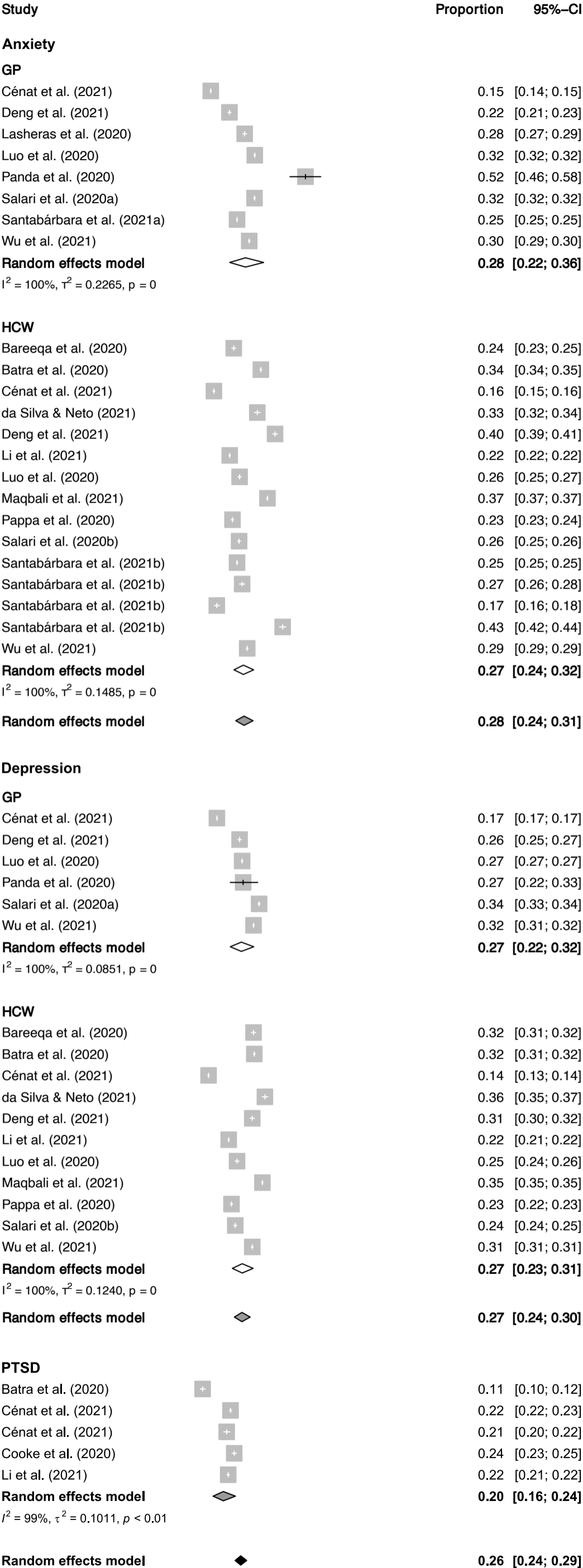

Supplement: Supplementary file 4 [file Image_2.PDF]

**Psychophysiological stress**

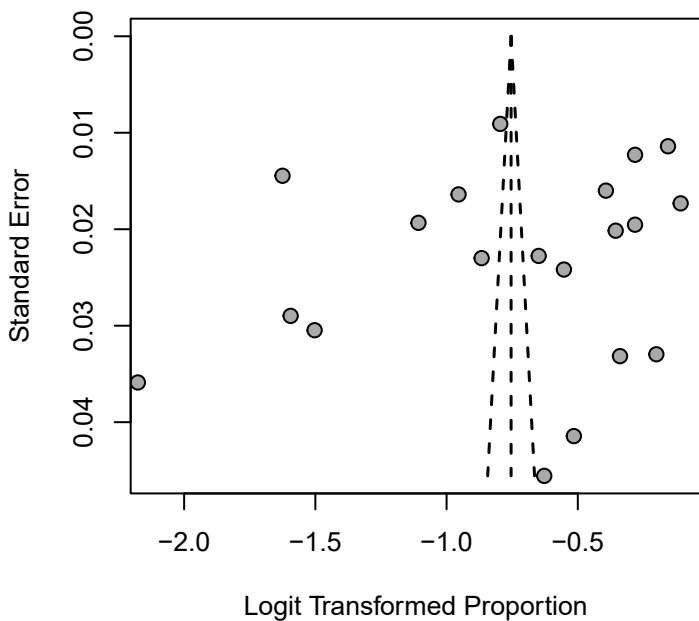

**Psychopathology**

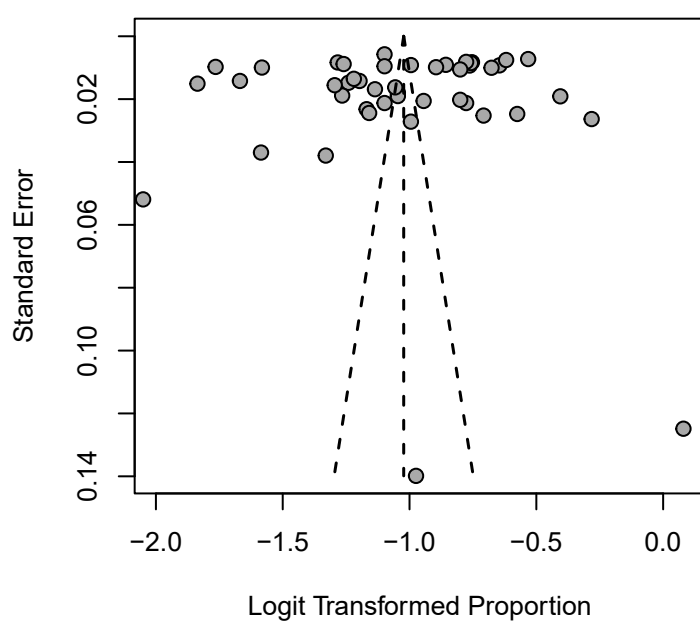

**Anxiety**

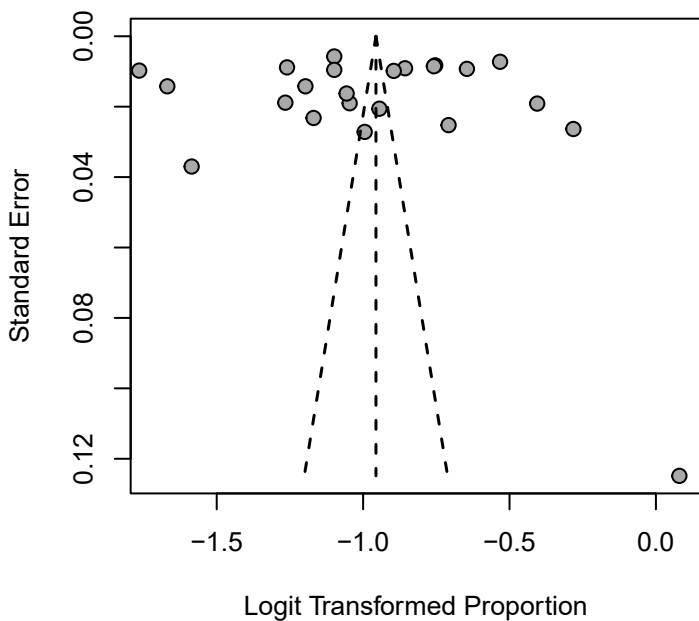

**Depression**

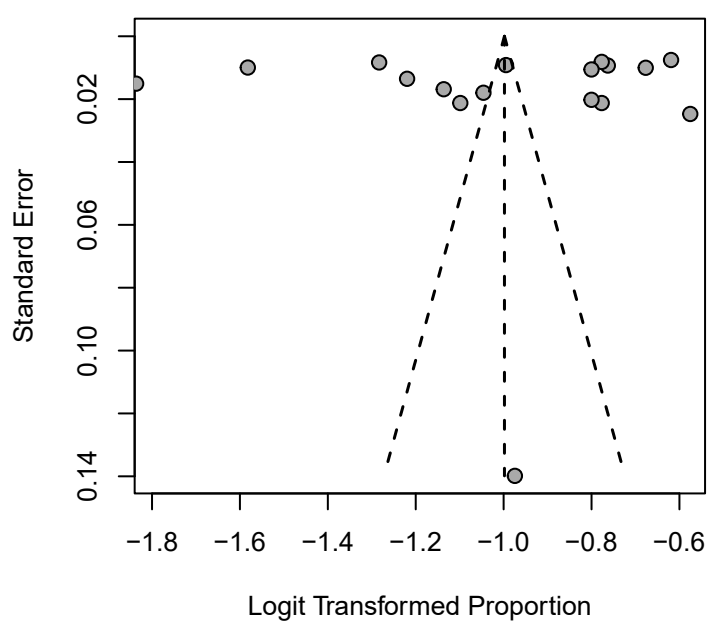

Supplement: Supplementary file 5 [file Image_3.PDF]
